# Some Dental Facts

**Published:** 1919-09

**Authors:** 


### SOME DENTAL FACTS

A match-stick is usually thrown away as soon as the cigarette is lit, but if a few of the "strike-anywhere" kind are saved they come in well. If one is chucked in a crayon, or lead holder, to carry a wrap of cotton, with which to apply iodine to the gums, it saves a steel instrument. Then if half of one is chucked in right-angle and trimmed to a point it will be found to beat anything now in existence to carry pumice in polishing teeth. It will get around the roots as no other thing will—and by the way, the pumice should be mixed with iodine saturated in benzol. If a match-stick is cut bias, just back of the charred end, it is good for carrying soldering fluid to gold before soldering.

[2] Hess, A. F.: Subacute and Latent Infantile Scurvy, J. A. M. A. 68: 235 (Jan. 27) 1917.

A wedge of compressed match-stick is much more endurable to the patient in wedging teeth than a piece of rubber and is excellent to put in the V space at the gum line in filling approximal cavities with amalgam.

Save some aluminum box tops also. Crescent-shaped pieces should be made from them to imbed in rubber plates across the median line to prevent splitting or cracking. And good lingual bars from lower plates can be made from an antiphlogistine spatula.

If cotton "drawings," size of the middle finger, are obtained from a cotton mill, boiled in soda and water, dried, and cut into suitable lengths and at the time of use wrapped in a square of bibulous paper, they make softer and more adapted rolls than any on the market and they are cheaper.

Some patients have an abhorrence to a dry napkin—if small squares of paraffine paper are used to lay across the tongue, discomfort to them is prevented. This paper is water-proof also. Lower impression cups can be used for upper impressions with plaster as handily as with regular upper cups, if first an impression is taken with beeswax, chilled and then the plaster poured into it, When the plaster cast is made for a full upper set, locate in the mouth with a finger the depression of the posterior platine fossa—near the position of the wisdom teeth on each side—make a corresponding depression in the plaster cast. A plate made from such a cast will fit better and the protuberance will prevent food getting under it.

If a patient can not be seen after an impression and wax bite are taken before the set or sets for full upper and lower jaws are inserted, after the plaster casts are put on the articulator, move the upper part of the articulator forward 1/8 to 3-16 of an inch, according to judgment, and 95 per cent. of cases will have the bite just right—for the inclination in biting the wax is to throw the lower jaw forward that much.

Now after using the plaster the best thing to cleanse the bowl from it is to use a round paint brush 1 1/4 inches in diameter with the water.

Wheels cut from 3-16 inch thick rubber belting are the most efficient carriers of pumice in dressing down and removing scratches from rubber plates and gold crowns—the layer of cotton fiber holds the pumice while the rubber layer rubs it in.—*Dental Facts.*

